# Immunomodulator Clarithromycin Enhances Mucosal and Systemic Immune Responses and Reduces Re-Infection Rate in Pediatric Patients with Influenza Treated with Antiviral Neuraminidase Inhibitors: A Retrospective Analysis

**DOI:** 10.1371/journal.pone.0070060

**Published:** 2013-07-17

**Authors:** Wakako Shinahara, Etsuhisa Takahashi, Takako Sawabuchi, Masaru Arai, Nobuo Hirotsu, Yoshio Takasaki, Shizuo Shindo, Kyoko Shibao, Takashi Yokoyama, Kiyoshi Nishikawa, Masahiro Mino, Minako Iwaya, Yuji Yamashita, Satoshi Suzuki, Dai Mizuno, Hiroshi Kido

**Affiliations:** 1 Division of Enzyme Chemistry, Institute for Enzyme Research, The University of Tokushima, Tokushima, Japan; 2 Arai Clinic, Chichibu, Japan; 3 Hirotsu Clinic, Kawasaki, Japan; 4 Takasaki Children's Clinic, Fukuoka, Japan; 5 Shindo Children's Clinic, Fukuoka, Japan; 6 Shibao Clinic, Fukuoka, Japan; 7 Yokoyama Children's Clinic, Kasuga, Japan; 8 Nishikawa Clinic, Zentsuji, Japan; 9 Mino Children's Clinic, Kannonji, Japan; 10 Iwaya Children's Clinic, Fukuoka, Japan; 11 Yamashita Children's Clinic, Maebaru, Japan; 12 Nagoya City Jouhoku Hospital, Nagoya, Japan; Albany Medical College, United States of America

## Abstract

**Background/Aims:**

Treatment with antiviral neuraminidase inhibitors suppresses influenza viral replication and antigen production, resulting in marked attenuation of mucosal immunity and mild suppression of systemic immunity in mice. This study investigated the effects of immunomodulator clarithromycin (CAM) supplementation on mucosal and systemic immunity in pediatric patients with influenza treated with neuraminidase inhibitors.

**Methods:**

A retrospective, non-randomized case series study was conducted among five treatment groups of 195 children aged 5.9±3.3 years infected with influenza A in 2008/2009 season. The five treatment groups were oseltamivir (OSV), zanamivir (ZNV), OSV+CAM, ZNV+CAM and untreated groups. Anti-viral secretory IgA (S-IgA) levels in nasal washes and IgG levels in sera were measured. The re-infection rate was analyzed among the same five treatment groups in the 2009/2010 season.

**Results:**

Treatment of influenza with OSV and ZNV for 5 days attenuated the induction of anti-viral S-IgA in nasal washes and anti-viral IgG in serum, compared with the untreated group. The combination of CAM plus OSV or ZNV boosted and restored the production of mucosal S-IgA and systemic IgG. The re-infection rates in the subsequent season were significantly higher in the OSV and ZNV groups than the untreated, while CAM+OSV and CAM+ZNV tended to reduce such rate.

**Conclusions:**

CAM restored the attenuated anti-viral mucosal and systemic immunity and reduced the re-infection rate in the subsequent year in pediatric patients with influenza treated with OSV and ZNV.

## Introduction

Influenza is a worldwide public health problem, particularly with emerging new strains to which vaccines are ineffective, limited, or unavailable. The antiviral neuraminidase inhibitors oseltamivir (OSV) and zanamivir (ZNV) are important treatment options for seasonal influenza infections [1], [2], and are being stockpiled in many countries as part of their pandemic response planning. These inhibitors impair the release of new influenza virions from infected cells by blocking the actions of viral neuraminidases [2], resulting in effective suppression of viral RNA replication and viral antigen production. In contrast to the therapeutic effects of OSV, we reported recently that OSV significantly suppressed the production of mucosal antigen (Ag)-specific secretory IgA (S-IgA) antibody and Ag-specific IgA-forming cells in the mouse airway, probably due to the suppressed viral antigen production, but it did not seriously suppress the production of systemic anti-viral IgG and IgG-forming cells in the spleen [3].

In order to prevent complications and aggravation of the flu symptoms, it is not uncommon, in Japan, to prescribe clarithromycin (CAM) developed by modification of erythromycin [4], an immunomodulator macrolide antibiotic [5]–[8] with antiviral activities [9], [10], in combination with OSV or ZNV. In this regard, we previously reported that administration of CAM in influenza A virus (IAV)-infected mice suppressed tumor necrosis factor alpha production and augmented interleukin-12 production in the blood [11], [12], resulting in alleviation of the flu symptoms, while oral treatment with OSV attenuated the induction of respiratory anti-IAV specific secretory IgA (S-IgA) immune responses [3]. Furthermore, we have verified in IAV-infected children that oral CAM augments the nasopharyngeal mucosal immune responses, while OSV suppresses the production of mucosal anti-IAV S-IgA [13]. Of interest, we have also reported that 75% of patients treated with the combination of CAM and OSV show increases in S-IgA production to levels similar to those seen in patients treated with CAM alone and untreated patients. In addition, we recently determined the molecular mechanisms responsible for the enhanced induction of mucosal IgA class switching recombination in CAM-treated mice [14]. The obtained data indicated that CAM significantly enhances the expression levels of B-cell-activating factor of the tumor necrosis factor family (BAFF) molecule on mucosal dendritic cells as well as those of activation-induced cytidine deaminase and Iμ-Cα transcripts on B cells [14]. The results indicated that CAM enhances S-IgA production through the induction of IgA class switching recombination in IAV-infected mice.

In previous clinical studies [13] on the immunomodulatory and boost effects of CAM on the nasopharyngeal mucosal immune response in pediatric patients with influenza treated with OSV, several questions remain to be answered: (i) Do antiviral neuraminidase inhibitors other than OSV, such as ZNV, an orally inhaled powder, also suppress the adaptive respiratory S-IgA response? (ii) Do the antiviral neuraminidase inhibitors also affect serum IgG responses in pediatric influenza? (iii) Do antiviral neuraminidase inhibitors, with and without CAM, affect the rate of future influenza virus re-infection? The present retrospective and non-randomized case series study was conducted to provide answers to these questions in 195 children infected with IAV. We report here that treatment with ZNV suppressed airway mucosal immunity and systemic immunity in pediatric influenza in a manner similar to OSV. The addition of CAM induced a mild boost and tended to restore the suppressed mucosal anti-viral S-IgA response in the OSV- and ZNV-treated patients, and also boosted serum IgG response, with a significant improvement in anti-IAV-specific IgG production in the ZNV-treated group. In addition, CAM tended to decrease, albeit insignificantly, the re-infection frequency in the OSV- and ZNV-treated groups.

## Methods

### Ethics Statement

After explanation of the purpose of this clinical study, written informed consent was obtained from each parent of pediatric patients for enrollment in the study and for the use of stored nasopharyngeal aspirates and blood for quantitative analyses of anti-IAV antibodies. Permission to perform clinical studies and ethical approval of the study protocol were granted by the Ethics Committee of Tokushima University Hospital (Permit Number, #463). The study was conducted under the supervision of the pediatricians involved (MA, NH, YT, SS, KS, TY, KN, MM, MI, YY and SS), and parents were advised of risks, benefits and the right to withdraw their children from further involvement in the study at any point without repercussions. All data, particularly patient identification data, were physically and electronically secured throughout the study.

### Study population

The study subjects were 195 children (age, 5.9±3.3 years, mean±SD, range, 0–14 years), who were infected with IAV between October 2008 through March 2009 in 11 Pediatric Clinics and Children's Hospitals in the mid-west region of Japan. A descriptive survey study on re-infection was conducted for the same children from October through March of 2009/2010. The inclusion criteria were the followings: patients who presented to the Pediatric Clinics and Children's Hospitals and diagnosed with the rapid diagnosis Espline Influenza A&B-N kit (Fujirebio Inc., Tokyo, Japan) and whose treatment was initiated within 48 hours of the onset of fever. Patients with congenital defects and those with co-morbid chronic diseases were excluded. Since the number of Japanese infected with influenza B in the 2008/2009 season was not large [15], and the antigen of influenza B/Victoria lineage prevailing in the season was not commercially available for the analysis of antibody titers, statistical analysis was conducted only on data of patients who presented with IAV.

### Treatment regimens

Patients diagnosed with IAV infection were divided into five groups according to the prescription of each pediatrician involved: the no-treatment group (n = 68), the OSV group (70 patients treated orally twice daily with OSV at 2 mg/kg body weight for 5 days), OSV+CAM group (20 patients treated orally twice daily with OSV at 2 mg/kg body weight for 5 days plus oral CAM at 5.0–7.5 mg/kg body weight for 5 days), the ZNV group (27 patients older than 4 years treated twice daily with orally inhaled ZNV powder at 10 mg for 5 days) and ZNV+CAM group (10 patients treated twice daily with orally inhaled ZNV powder at 10 mg for 5 days plus oral CAM at 5.0–7.5 mg/kg body weight for 5 days). There were no outbreaks of *Mycoplasma* or *Chlamydia* at the time of the study. All patients were followed for 5 days.

### Collection of biological samples

All children suspected clinically to have influenza underwent both nasopharyngeal aspiration and serum collection. Nasopharyngeal aspiration was conducted on each nostril for 1 minute, through a silicon tube, and the aspirate collected in a centrifuge tube connected to an evacuator, as described previously [16], [17]. The isolated specimens were immediately cooled on ice, homogenized by sonification for 20 seconds on ice, in a model 250, 20% duty, 2-cycle Sonifier® (Branson Ultrasonics Co., Danbury, CT), and the insoluble materials were removed by centrifugation at 2000× *g* for 5 minutes at 4°C. The supernatants of nasopharyngeal specimens and serum were stored at −30°C until use.

### Enzyme-linked immunosorbent assay (ELISA)

The concentrations of total IgA, IgG and anti-IAV-specific S-IgA in nasopharyngeal specimens and anti-IAV-specific IgG in sera were measured by ELISA, as described previously [13], [17]. For measurement of anti-IAV-specific antibody, the prevalent IAV strains were selected as coating ELISA antigens: In the 2008/2009 flu season before May 2009, IAV/Brisbane/59/2007(H1N1)-like and IAV/Uruguay/716/2007(H3N2)-like subtypes were prevalent in Japan [15]. Since the affinity purified human anti-IAV-specific S-IgA and IgG standards for each IAV subtypes are not commercially available, the concentrations of anti-IAV-specific antibody in the nasopharyngeal specimens and sera were determined from the standard regression curves with human IgA and IgG of known concentrations in a human IgA and IgG quantitation kits (Bethyl Laboratories Inc., Montgomery, TX). The relative values of anti-IAV-specific S-IgA and IgG were expressed as units (U); one U of each anti-IAV-specific S-IgA and IgG was determined from the regression curves as the point corresponding to 1 µg of human IgA and 1 mg of human IgG detected in the assay system, respectively, as described previously [13], [17]. Since the concentration of nasal wash samples varies widely between individuals depending on the aspiration efficiency and patient age, the concentration of anti-IAV-specific S-IgA (U/mL) was normalized by the amount of protein (mg/mL). Since there was no significant variability in serum protein concentrations, the row values of anti-IAV-specific IgG concentrations (U/mL) were used. The protein concentrations in the nasopharyngeal specimens were measured using a bicinchoninic acid protein assay reagent kit (Pierce, Rockford, IL).

### Statistical analysis

Results are presented as the median (interquartile range), or numbers (%) of observations. The S-IgA levels in nasopharyngeal specimens of the different patient groups were compared by the Mann-Whitney U-test and Wilcoxon signed-rank test. Between group comparisons of disease symptoms were made using Fisher's exact test with the Bonferroni correction. A *P* value <0.05 was considered statistically significant.

## Results

### Patient characteristics

Table 1 lists the characteristics of patients of the five groups. The most common features of influenza were fever (defined as body temperature ≥38°C), sore throat, cough, nasal discharge, headache and body aches. About half (48.6–64.7%) of the patients in each group had received vaccination before the onset of the influenza season and no significant differences in the values were observed among the five groups. The prevalence of disease signs and symptoms at admission to hospital was similar among the five groups. The time between onset of illness and initial examination ranged from 1.0 to 1.9 days with a mean value of 1.7±0.6 days.

**Table 1:** Patient characteristics.

	All patients	No Treatment	OSV	OSV+CAM	ZNV	ZNV+CAM
	(n = 195)	(n = 68)	(n = 70)	(n = 20)	(n = 27)	(n = 10)
Age, years, (range)*	5.9±3.3 (0–14)	7.3±3.9 (0–14)	4.2±2.7 (0–10)	5.1±2.1 (1–9)	7.0±1.6 (5–9)	6.9±2.0 (4–9)
Time between onset illness and initial examination (days)*	1.7±0.6	1.6±0.7	1.7±0.6	1.9±0.6	1.6±0.6	1.0±0.7
Previous vaccination (%)	112(57.4)	44(64.7)	34(48.6)	12(60.0)	17(63.0)	5(50.0)
Fever (%)	180(95.2)	63(92.6)	68(100)	16(88.9)	25(96.2)	8(88.9)
Sore throat (%)	68(35.4)	24(35.3)	20(29.9)	4(20.0)	15(55.6)	5(50.0)
Cough (%)	173(88.7)	64(94.1)	61(87.1)	16(80.0)	23(85.2)	9(90.0)
Nasal discharge (%)	177(90.8)	67(98.5)	64(91.4)	17(85.0)	20(74.1)	9(90.0)
Headache (%)	70(36.6)	26(38.2)	20(30.3)	4(20.0)	14(51.9)	6(60.0)
Body aches (%)	54(28.7)	16(23.5)	24(37.5)	3(15.8)	8(29.6)	3(30.0)

*Data are mean±SD.

### Effects of OSV and ZNV on nasopharyngeal antiviral-S-IgA production and serum antiviral-IgG in patients treated with or without CAM

Table 2 summarizes the levels of anti-IAV(H1N1)- and (H3N2)-specific S-IgA (U/mg protein) and total S-IgA (µg/mg protein) before and 5 days after treatment with OSV and ZNV, with or without CAM. In the control (no treatment) group, significant increases in the concentrations of anti-IAV-specific S-IgA against subtypes H1N1 (*P*<0.05) and H3N2 (*P*<0.01) were observed at 5 days after infection. The percentages of patients with greater than or equal (≥1-fold) to baseline titer before treatment and greater than or equal to a four-fold increase (≥4-fold) from baseline titer in the no-treatment group against subtype H1N1 were 61.5 and 26.2, respectively, and against subtype H3N2 were 69.2 and 15.4, respectively. Treatment with OSV and ZNV significantly suppressed the percentage of patients with ≥1-fold of baseline titer against both H1N1 and H3N2 subtypes, compared with the values in the no-treatment group. However, co-administration of CAM and OSV boosted S-IgA induction and increased the percentage of patients with ≥1-fold of baseline titer from 42.9 to 65.0 against H1N1 and from 42.9 to 70.0 against H3N2 (*P* value versus OSV group for H1N1: *P*<0.06 to <0.09, H3N2: <0.05). Co-administration of CAM and ZNV resulted in mild restoration of the suppressed percentage of patients with ≥1-fold of baseline titer from 37.0 to 50.0 against H1N1 and from 48.1 to 60.0 against H3N2, although the differences between the two were not statistically significant. There were no significant differences in the percentages of patients with ≥4-fold of baseline S-IgA titer among the five treatment groups.

**Table 2:** Changes in anti-IAV-specific S-IgA production in untreated patients and patients treated with OSV and ZNV for 5 days, with or without CAM.

Treatment	n	S-IgA concentration						Percentage of patients with ≥1-fold and ≥4-fold increases in anti-IAV-specific S-IgA concentration during treatment			
		Anti-IAV-specific S-IgA (U/mg protein)				Total S-IgA (µg/mg protein)					
		H1N1		H3N2				H1N1		H3N2	
		Before	After	Before	After	Before	After	≥1-fold (After/before)	≥4-fold (After/before)	≥1-fold (After/before)	≥4-fold (After/before)
No treatment	65	2.3 (0.5–5.7)	3.1 (1.2–7.8)*	2.2 (0.6–3.8)	3.1 (1.0–6.7)¶	120.6 (90.3–177)	143.5 (118–204)*	61.5	26.2	69.2	15.4
OSV	70	1.3 (0.6–5.9)	1.2 (0.4–5.0)	1.0 (0.4–2.8)	1.1 (0.5–3.1)	122.8 (79.1–147)	128.0 (82.8–163)	42.9§	18.6	42.9^‡^	14.3
OSV+CAM	20	0.9 (0.3–1.4)	1.9 (0.3–12.3)*	0.7 (0.2–1.7)	1.5 (0.3–7.6) ¶	121.6 (74.1–153)	134.1 (98.9–214)*	65.0#	30.0	70.0^†^	20.0
ZNV	27	3.8 (1.7–12.3)	4.5 (0.5–15.8)	2.0 (1.4–3.8)	2.6 (0.5–7.8)	109.7 (86.8–181)	149.8 (101–216)	37.0§	18.5	48.1§	11.1
ZNV+CAM	10	2.0 (1.6–7.1)	6.3 (2.2–19.5)	1.7 (1.2–2.4)	4.3 (1.8–11.6)	143.3 (104–202)	173.2 (151–207)	50.0	30.0	60.0	30.0

Data are median (interquartile range). Before and after denote before and after treatment, respectively.

*P<0.05,

¶ *P*<0.01, versus before treatment for the same parameter (Wilcoxon signed-ranks test).

§ *P*<0.05, versus the respective no treatment (Fisher's exact test). ^‡^ *P*<0.01, versus the respective no treatment (Fisher's exact test).

# *P*<0.06 to <0.09, versus the OSV group (Fisher's exact test). ^†^ *P*<0.05, versus the OSV group (Fisher's exact test).

Table 3 lists the levels of serum anti-IAV(H1N1)- and (H3N2)-specific IgG (U/mL) and total IgG (mg/mL) before and 5 days after treatment of IAV infection with OSV and ZNV, with or without CAM. Significant increases were noted in serum levels of anti-IAV-specific IgG in all groups at 5 days after treatment, except those against H1N1 in the OSV and ZNV groups. In particular, ZNV treatment significantly reduced the percentage of patients with ≥1-fold of baseline titer against H1N1 compared with the no-treatment group, from 73.5 to 40.9 (*P*<0.01), in a manner similar to that noted in mucosal S-IgA responses. In addition, the combination of CAM plus ZNV significantly increased the percentage of patients with ≥1-fold of baseline titer against H1N1 (*P*<0.01), though the increase against H3N2 was marginal, (*P*<0.06 to <0.09), compared with ZNV alone. There were no significant differences in the percentages of patients with ≥4-fold of baseline IgG titer among the five treatment groups.

**Table 3:** Changes in anti-IAV-specific IgG production in untreated patients and patients treated with OSV and ZNV for 5 days, with or without CAM.

Treatment	n	IgG concentration						Percentage of patients with ≥1-fold and ≥4-fold increases in anti-IAV-specific IgG concentration during treatment			
		Anti-IAV-specific IgG (U/mL)				Total IgG (mg/mL)					
		H1N1		H3N2				H1N1		H3N2	
		Before	After	Before	After	Before	After	≥1-fold (After/before)	≥4-fold (After/before)	≥1-fold (After/before)	≥4-fold (After/before)
No treatment	49	0.5 (0.2–0.9)	0.8 (0.4–1.5)¶	0.4 (0.2–0.7)	0.8 (0.3–1.4)¶	19.3 (10.9–48.3)	17.7 (10.2–28.1)*	73.5	30.6	73.5	34.7
OSV	52	0.2 (0.03–0.7)	0.5 (0.06–1.0)	0.2 (0.04–0.5)	0.4 (0.06–1.1)¶	13.2 (9.1–17.6)	11.8 (8.6–17.4)	63.5	21.2	75.0	32.7
OSV+CAM	14	0.5 (0.3–0.6)	0.9 (0.5–1.4)¶	0.5 (0.2–0.5)	0.6 (0.4–1.2)*	14.1 (10.1–21.1)	12.0 (9.6–16.7)	78.6	21.4	85.7	14.3
ZNV	22	0.6 (0.3–1.1)	0.6 (0.4–1.1)	0.3 (0.2–0.5)	0.5 (0.4–0.9)¶	11.9 (8.3–31.4)	15.3 (9.4–28.4)	40.9‡	13.6	68.2	18.2
ZNV+CAM	8	0.3 (0.2–0.6)	0.9 (0.2–1.8)*	0.4 (0.2–0.7)	1.4 (0.4–2.6)*	15.4 (10.2–22.0)	12.0 (8.9–14.9)	100.0†	25.0	100.0#	50.0

Data are median (interquartile range). Before and after denote before and after treatment, respectively.

*P<0.05,

¶ *P*<0.01, versus before treatment for the same parameter (Wilcoxon signed-ranks test).

‡ *P*<0.01, versus the respective no treatment (Fisher's exact test).

# *P*<0.06 to <0.09, versus the ZNV group (Fisher's exact test).

† *P*<0.01, versus the ZNV group (Fisher's exact test).

Table 4 compares the prevalence of disease manifestations at day 5 after treatment. Significant improvements were noted in the prevalence of cough in the OSV+CAM group, compared with the OSV group (*P*<0.05), and nasal discharge in the ZNV+CAM group, compared with the ZNV group (*P*<0.05). However, there were no significant differences in the effects of various treatments on the other listed symptoms among the five treatment groups.

**Table 4:** Rates of improvement of clinical symptoms after 5 days of no treatment and treatment with OSV, OSV+CAM, ZNV and ZNV+CAM.

Improvement (%)	Fever	Sore throat	Cough	Nasal discharge	Headache	Body aches
No treatment (n = 68)	94.1 (64/68)	92.0 (23/25)	35.4 (23/65)	41.5 (27/65)	96.0 (24/25)	100 (16/16)
OSV (n = 70)	94.3 (66/70)	68.2 (15/22)	31.3 (20/64)	39.4 (26/66)	100 (21/21)	100 (26/26)
OSV+CAM (n = 20)	95.0 (19/20)	80.0 (4/5)	58.8* (10/17)	61.1 (11/18)	100 (4/4)	100 (4/4)
ZNV (n = 27)	96.3 (26/27)	93.3 (14/15)	50.0 (12/24)	37.5 (9/24)	86.7 (13/15)	87.5 (7/8)
ZNV+CAM (n = 10)	100 (10/10)	100 (5/5)	60.0 (6/10)	77.8¶ (7/9)	100 (6/6)	100 (3/3)

Data are percentage of patients who reported disappearance of symptoms per patients with symptoms at the start of treatment.

* *P*<0.05, versus OSV (Fisher's exact test).

¶ *P*<0.05, versus ZNV (Fisher's exact test).

### Frequency of re-infection in subsequent year

**Figure 1:**
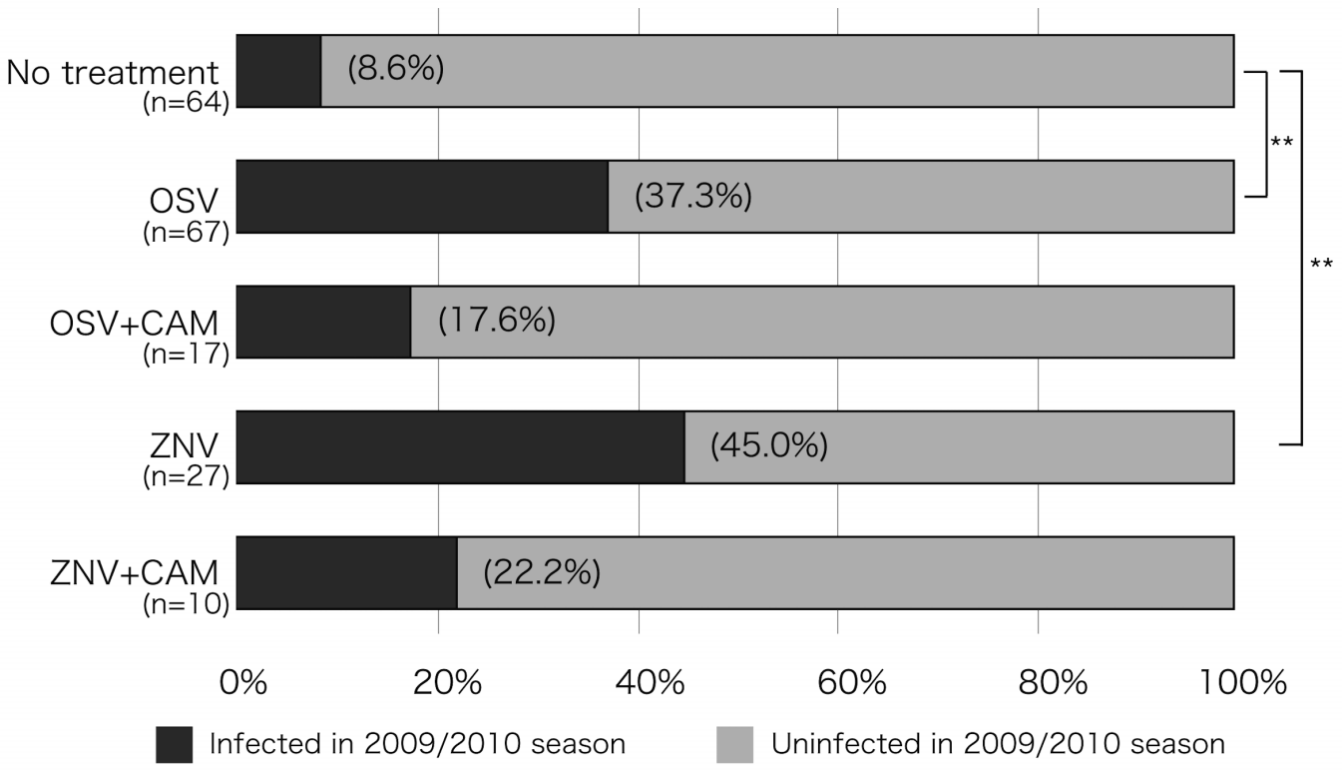
Re-infection rate in 2009/2010 season. The re-infection rate in 2009/2010 season in children who were infected with IAV during the 2008/2009 season and either untreated or treated with OSV, OSV+CAM, ZNV and ZNV+CAM. Data show the percentage of infected children in each group. * *P*<0.05, * * *P*<0.01, versus no treatment (Fisher's exact test with Bonferroni correction).

Based on the low level of acquired mucosal anti-IAV-specific S-IgA after infection, patients were at risk of re-infection in the subsequent year, particularly those treated with OSV or ZNV. Figure 1 lists the percentages of re-infected individuals according to the treatment received in the preceding year. The IAV pH1N12009 was the predominant circulating virus in Japan with a peak during October-December of 2009. Even under the spread of a new virus subtype in the 2009/2010, only 8.6% of the children of the no-treatment group were re-infected. However, the proportions of children treated the previous year with OSV and ZNV who developed re-infection in 2009–2010 were significantly higher at 37.3% and 45.0%, respectively (*P*<0.01), than those of the no-treatment group. The combination treatment of CAM plus OSV and CAM plus ZNV tended to reduce the re-infection rate to 17.6% and 22.2%, respectively, albeit insignificantly.

## Discussion

The main findings of the present study were the following: (i) Treatment with antiviral neuraminidase inhibitors, OSV and ZNV, tended to suppress the production of respiratory anti-IAV-specific S-IgA as well as systemic anti-IAV-specific IgG in pediatric patients with influenza. (ii) The combination treatment of CAM plus OSV or ZNV mildly or significantly enhanced the production of anti-IAV S-IgA in the nasopharyngeal specimens and/or anti-IAV IgG in sera and tended to restore the suppressed local mucosal and systemic immunity observed with antiviral inhibitor agents. (iii) The rates of IAV re-infection in the subsequent year were significantly higher for the OSV and ZNV groups than the control group, whereas the combination of CAM plus OSV or ZNV tended to reduce such rate.

There is general agreement that the first line of host defense against infection is mucosal immunity, particularly nasopharyngeal immunity, which constitutes a major component of the immunological humoral and cell-mediated responses in the upper and lower respiratory tracts [18], [19]. However, the currently available intramuscularly and subcutaneously-injected influenza vaccines predominantly induce systemic IgG but not S-IgA and weak cellular immunity in the airway mucosa [18]–[21]. In fact, the levels of anti-IAV S-IgA relative to the total sIgA were low in nasal washes of all influenza patients on admission, whereas serum levels of anti-IAV IgG levels varied widely, probably reflecting the history of infection and vaccination in these individuals [13], [17]. Treatment of pediatric influenza with OSV or ZNV for 5 days significantly suppressed acquired anti-IAV S-IgA levels in nasal washes (Table 2) and these changes may also explain the higher frequency of re-infection in the OSV and ZNV groups in the subsequent year (Figure 1). The results may be supported by previous findings that mucosal S-IgA is primarily involved in cross-protection of the mucosal surface against variant IAV infection, and the mechanism of broad-spectrum cross-protection could be explained by the wide-range cross-reactivity of S-IgA [22]–[25].

The suppressive effects of OSV and ZNV on mucosal anti-IAV S-IgA levels, probably due to diminution of viral antigen production by anti-viral neuraminidase inhibitors, seem to be ameliorated by co-administration of CAM. CAM boosted mucosal and/or systemic immunity and tended to increase the levels of anti-IAV S-IgA in nasal washes and IgG in serum in the OSV- and ZNV-treated patients (Tables 2 and 3). This effect of CAM resulted in an increase in the percentage of patients with ≥1-fold of baseline titer before treatment, particularly S-IgA in the OSV-treated patients and IgG in the ZNV-treated patients. Although patients of the ZNV group were slightly older (about 2 years) than those of the OSV group, because of age limitation of oral inhalation of ZNV powder, the observed effects of ZNV on mucosal and systemic immunity were similar to those of OSV with or without CAM. The effects of OSV and ZNV with or without CAM could be clearer in naïve children with low or undetectable pre-existing immunological memory. The present results emphasize the need to study the effects of CAM in adult patients with pre-existing immunological memory and elderly patients with low immunological responses.

Nasopharyngeal-associated lymphoreticular tissue is known as the production site of nasal S-IgA, where IgA-committed B cells undergo class switching. Subsequently, IgA-committed B cells migrate to mucosal effector tissues including the nasal passages [26]. We reported recently that CAM enhances IgA class switching recombination through upregulation of BAFF in mucosal dendritic cells and activation-induced cytidine deaminase in B cells [14]. The present clinical results add support to these early studies.

In conclusion, the present study showed that CAM boosts and tends to restore the suppressed mucosal and/or systemic immunity in pediatric patients with influenza treated with OSV and ZNV.
