# Correction: Immunomodulator Clarithromycin Enhances Mucosal and Systemic Immune Responses and Reduces Re-Infection Rate in Pediatric Patients with Influenza Treated with Antiviral Neuraminidase Inhibitors: A Retrospective Analysis

**DOI:** 10.1371/journal.pone.0104573

**Published:** 2014-07-31

**Authors:** The PLOS ONE Staff

There are errors in Figure 1 and in the last two sentences of the fourth paragraph of the Results section.

The labeling of the number of patients and the re-infection rates are incorrect in Figure 1. Please see the corrected Figure 1 here.

**Figure 1:**
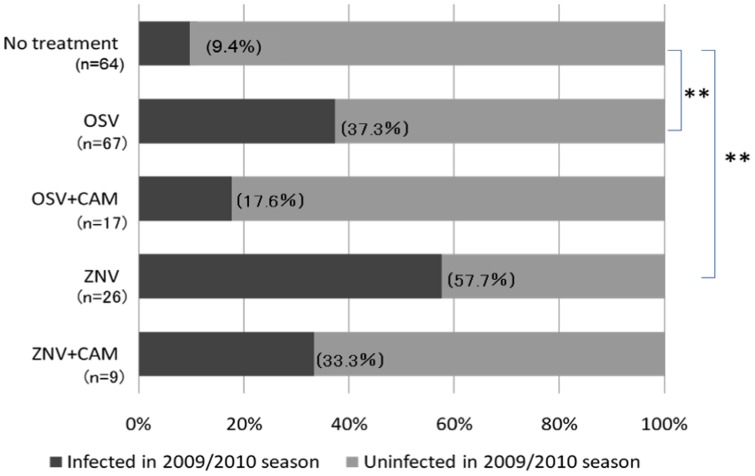
Re-infection rate in 2009/2010 season. The re-infection rate in 2009/2010 season in children who were infected with IAV during the 2008/2009 season and either untreated or treated with OSV, OSV+CAM, ZNV and ZNV+CAM. Data show the percentage of infected children in each group. *P<0.05, * *P<0.01, versus no treatment (Fisher's exact test with Bonferroni correction).

There are errors in the last two sentences of the fourth paragraph of the Results section. The corrected sentence should read: “However, the proportions of children treated the previous year with OSV and ZNV who developed re-infection in 2009-2010 were significantly higher at 37.3% and 57.5%, respectively (P<0.01), than those of the no-treatment group. The combination treatment of CAM plus OSV and CAM plus ZNV tended to reduce the re-infection rate to 17.6% and 33.3%, respectively, albeit insignificantly.”
